# Spectral Discrimination of the Invasive Plant *Spartina alterniflora* at Multiple Phenological Stages in a Saltmarsh Wetland

**DOI:** 10.1371/journal.pone.0067315

**Published:** 2013-06-27

**Authors:** Zu-Tao Ouyang, Yu Gao, Xiao Xie, Hai-Qiang Guo, Ting-Ting Zhang, Bin Zhao

**Affiliations:** 1 Coastal Ecosystems Research Station of the Yangtze River Estuary, Ministry of Education Key Laboratory for Biodiversity Science and Ecological Engineering, Institute of Biodiversity Science, Fudan University, Shanghai, China; 2 Department of Environmental Sciences, University of Toledo, Toledo, Ohio, United States of America; University of Florida, United States of America

## Abstract

*Spartina alterniflora* has widely invaded the saltmarshes of the Yangtze River Estuary and brought negative effects to the ecosystem. Remote sensing technique has recently been used to monitor its distribution, but the similar morphology and canopy structure among *S. alterniflora* and its neighbor species make it difficult even with high-resolution images. Nevertheless, these species have divergence on phenological stages throughout the year, which cause distinguishing spectral characteristics among them and provide opportunities for discrimination. The field spectra of the *S. alterniflora* community as well as its major victims, native *Phragmites australis* and *Scirpus mariqueter*, were measured in 2009 and 2010 at multi-phenological stages in the Yangtze River Estuary, aiming to find the most appropriate periods for mapping *S. alterniflora*. Collected spectral data were analyzed separately for every stage firstly by re-sampling reflectance curves into continued 5-nm-wide hyper-spectral bands and then by re-sampling into broad multi-spectral bands – the same as the band ranges of the TM sensor, as well as calculating commonly used vegetation indices. The results showed that differences among saltmarsh communities’ spectral characteristics were affected by their phenological stages. The germination and early vegetative growth stage and the flowering stage were probably the best timings to identify *S. alterniflora*. Vegetation indices like NDVI, ANVI, VNVI, and RVI are likely to enhance spectral separability and also make it possible to discriminate *S. alterniflora* at its withering stage.

## Introduction

The Yangtze River Estuary is an important eco-region that covers the main part of Shanghai and a portion of Jiangsu province, China. There are more than 300 km^2^ of coastal saltmarshes in Shanghai alone that cover 22.5% of its total area and 95.7% of its total wetland area and provide more than 95% of its total value of ecosystem service ($7.3×10^9^ US/year) [1]. However, one recent event, i.e., the *Spartina alterniflora* (hereafter, *Spartina*) invasion, has caused profound impacts on the saltmarshes [2]. The *Spartina* invasion to the Yangtze River Estuary will likely converse mudflats to *Spartina* meadows, destroy shorebirds’ foraging habitats, bring negative impacts on endangered species, decrease abundance of native species, and cause ecosystem degradation and considerable economic loss and the expanding trend is still not controlled [3]. These negative impacts of *Spartina* invasion as well as its wide and dynamic distribution have posed an urgent task to get its spatial information accurately and timely in order to conserve native biodiversity and manage the land ecologically.

Techniques of both multi-spectral and hyper-spectral remote sensing have been used to monitor and map saltmarsh vegetation. However, to monitor invasive plant species, the ability to map vegetation at monospecific level is required and thus we have the problems such as different spectra for the same vegetation type, the same spectra for different vegetation types, and mixed pixels that should be minimized. Hyper-spectral sensors with contiguous and narrow bands are able to detect small and local variations which are likely masked within broad bands of multi-spectral data; thus, hyper-spectral data can increase the potential to discriminate vegetation species and have successfully mapped wetland monospecific vegetation covers [4], [5] for a long period of time. On the other hand, though the old generation multi-spectral data such as Landsat TM and SPOT imageries were reported to be insufficient for mapping monospecific covers in detailed wetland environments due to mixed pixels resulting from coarse spatial resolution [4], [6], the high spatial resolution sensors that were newly launched (e.g., QuickBird and IKONOS) and aerial photography offer opportunities to monitor monospecific covers in wetlands by achieving resolutions of meters or sub-meter [7].

In spite of all of this, there is a common problem for either hyper-spectral imagery or VHR (very high-resolution) imagery in that they are difficult and expensive to acquire [4], [8]. This proposes an issue that the collected remote sensing data coincides with a period when suitable spectral differences between different vegetation types are captured by remote sensors. Field spectral discrimination serves as an important way to find the differences existing in spectral signatures between plant species [9], [10]. On the other hand, spectral characteristics of vegetation change obviously with time affected by phenology, complicating the problem but also providing opportunities of spectral discrimination and remote sensing mapping (i.e., some phases must be more appropriate than others for mapping). Therefore, it is important to identify species by exploiting spectral differences at proper phenological stages [11]. Furthermore, spectral differences can be enhanced by using vegetation indices (VIs) that take advantage of vegetation reﬂectance contrasts between wavebands/wavelengths [12]. VIs based on multi-spectral broad bands are commonly used to enhance vegetation differentiation [13], [14], but as an influence of phonology, the effectiveness of different VIs may also change with time. Recently, a few studies have taken advantages of phenology for species discrimination [12], [15]. Nevertheless, few studies concern multi-phonological spectra of saltmarsh vegetation, especially identifying the widespread invasive species, perhaps because of the difficult accessibility of saltmarsh environments for multi-date investigations. However, such work could provide important information guiding future field spectra sampling and remote sensing image collection.

This study aims to find out the most appropriate periods and corresponding spectral zones for mapping *Spartina* by remote sensing in the Yangtze River Estuary. We hope the expected information would help collect, process, and classify images at suitable periods for dynamically monitoring *Spartina*. To achieve this, spectral analyses were applied at multi phonological stages by sampling spectral curves into hyper−/multi-spectral bands, calculating VIs, and quantifying the spectral differences at each pure band and VI for comparison. More specifically, the objectives of this study were: 1) to characterize the canopy spectral properties of dominant saltmarsh species in different phenological stages and 2) (the more important) to identify the most suitable phenological stages to discriminate invasive exotic *Spartina* and simultaneously determine the corresponding best separable hyper−/multi-spectral bands and VIs.

## Materials and Methods

### Ethics Statement

Our fieldwork in this study was approved by the Shanghai Chongming Dongtan National Nature Reserve. We did not sample any soil, plants, or animals out of the ecosystem in the work. Only reflectance spectra and photographs were collected with minimum disturbances.

### Study Area

**Figure 1:**
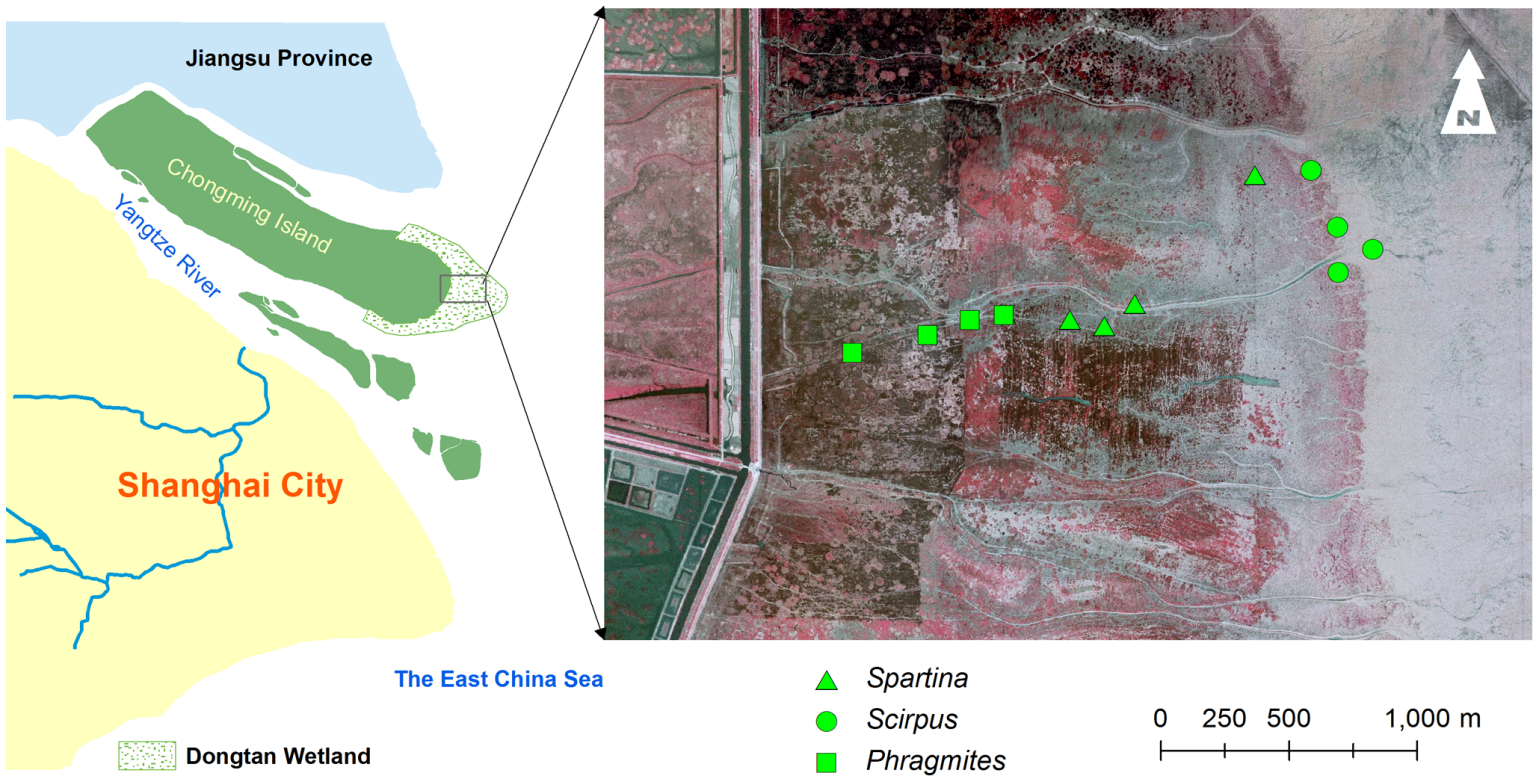
Map of the study area in Dongtan, Chongming Island. The locations of plant patches used for field spectral measurement was superimposed. The background image of the right frame is derived from an aerial photograph acquired in spring of 2006.

The study area is a broad saltmarsh at the eastern Chongming Island (Dongtan) (Fig. 1), the largest wetland in the Yangtze River Estuary of China. The shallow open waters, mudflats, and saltmarshes in Dongtan make an important habitat for shorebirds [3] and serve as an important stopover for migratory birds on the East Asian-Australian Flyway [16].

The saltmarshes of Dongtan were dominated by native plant species *Phragmites australis* (hereafter, *Phragmites*) and *Scirpus mariqueter* (hereafter, *Scirpus*) before the invasion of *Spartina*, and they create a good habitat and provide abundant food for various birds. However, the invasion of *Spartina* is seriously threatening the native ecosystem and coastal aquaculture, and the native species are rapidly being replaced by *Spartina* since it is introduced in 2001 for land reclamation [2].

The phenology of the three presently dominated species is somewhat different [17]. Generally, *Phragmites* germinates in early April, undergoes a rapid vegetative growth stage spanning from June to mid-August, flowers in mid-October, and senesces in late-November. *Spartina* emerges in early-May, grows rapidly from June to early-September, flowers in late-September, and dies in late-December. *Scirpus* has a relatively shorter lifespan than *Phragmites* or *Spartina*. It usually begins to grow in late-April, turns to flowering stage in mid-June, and starts withering process in early-September. These differences in phenology may greatly affect the distinctive spectral signatures between the species, making some periods during a year more likely than others to discriminate them using remote sensing technology.

### Spectral Data Acquisition

Spectral data of dominated plants was collected *in situ* using a UniSpec-DC Spectral Analysis System (PP System, Inc. Amesbury, MA, USA). This machine has a detection region of 310∼1 100 nm with optional FOVs of 3°, 6°, 12°, and 20°. The field work was initially planned to be done in 2009. However, limited by weather and tidal conditions, the data in some expected periods was missed in 2009. To fill the data gaps, the missing phenological stages were re-surveyed in 2010. In total, the spectra data was collected on 12 different dates at various phenological stages under clear weather between 10∶00 and 14∶00 (Table 1).

**Table 1:** Phenological description of major plants of the twelve data collection days.

Date	Phenological phases		
	*Phragmites*	*Spartina*	*Scirpus*
Jan. 14	Dormancy	Dormancy	Dormancy
Mar. 16	Dormancy	Dormancy	Dormancy
Apr. 17	Vegetative growth^1^(100–120 cm)	Dormancy	Germination(5–7 cm)
May 5	Vegetative growth^1^(140–160 cm)	Germination(30–50 cm)	Vegetative growth^1^(15–20 cm)
May 31	Vegetative growth^2^(180–200 cm)	Vegetative growth^1^(100–120 cm)	Vegetative growth^2^(50–60 cm)
Jul. 2	Vegetative growth^2^(180–200 cm)	Vegetative growth^2^(180–200 cm)	Flowering(50–60 cm)
Jul. 22	Vegetative growth^2^(180–200 cm)	Vegetative growth^2^(180–200 cm)	Development of fruit(50–60 cm)
Aug. 19	Vegetative growth^2^(180–200 cm)	Inflorescence(180–200 cm)	Maturity of fruit and seed(50–60 cm)
Sep. 20	Inflorescence(180–200 cm)	Flowering(180–200 cm)	Withering(50–60 cm)
Oct. 15	Flowering(180–200 cm)	Maturity of fruit and seed(180–200 cm)	Withering(50–60 cm)
Nov. 25	Withering(180–200 cm)	Withering(180–200 cm)	Dormancy
Dec. 22	Dormancy	Withering(180–200 cm)	Dormancy

Quantitative values in parentheses are plant height of corresponding plants estimated by vision. The difference between vegetative growth^1^ and vegetative growth^2^ is that stems elongate quickly in the former while little stem development and mainly development of harvestable vegetative part occurs in the latter. On January 14 and March 16, a large part of *Scirpu*s covers fell down due to tidal water and, thus, their canopy height was lower than the height given in this table.

**Figure 2:**
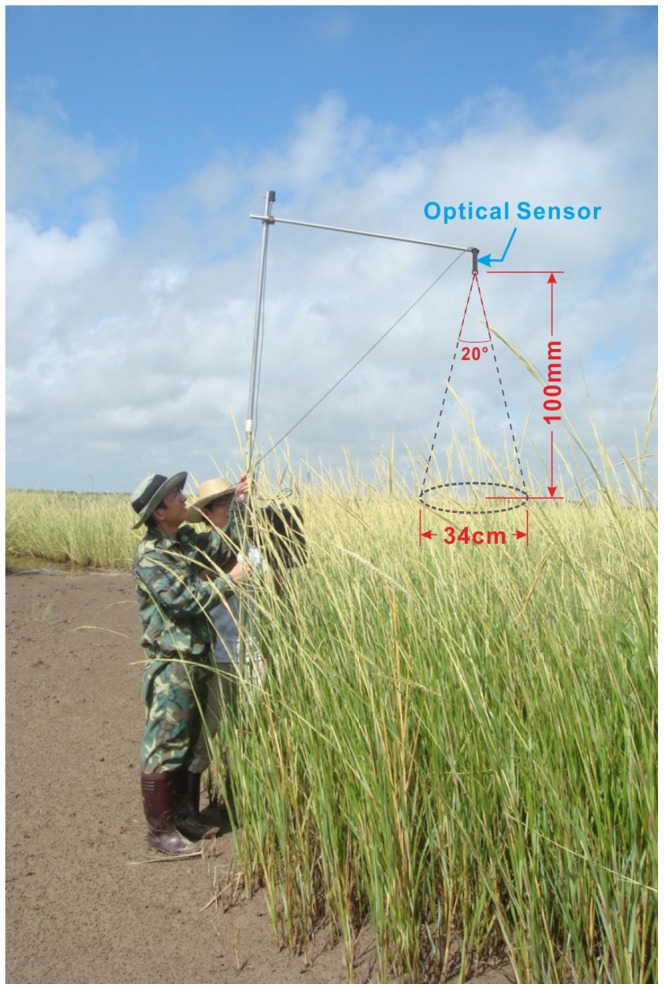
Field spectral measurement above the *Spartina* canopy.

Four patches (larger than 10 m×10 m) with similar vegetation height and coverage were selected for each plant species to decrease multi-factors interaction and influence (Fig. 1). Three random sites were taken in each patch (sometimes, only in three patches) for spectral data collection and 20 spectrometer measurements were taken from each site at nadir points. The sensor was placed above plant canopies at approximately 100 cm height using a 20°FOV optic, which resulted in approximately 0.34 m spatial resolution (Fig. 2). The spectral data were calibrated by a standard white reference and converted into reflectance data.

### Data Pre-analysis

Only the data between 400 nm and 900 nm were used for analysis due to extraneous noises at the extremes of the detection range. The sampled data of each surveyed day were analyzed separately following the same steps below.

First, the 20 measurements from each site were averaged as one reflectance curve sample, resulting in 9∼12 samples for each plant species. Second, the reflectance curves were divided into 100 consecutive 5 nm-wide hyper-spectral bands by taking the average from its original five wavelengths without losing much information (the raw spectral resolution is 3.3 nm). Moreover, reﬂectance data were averaged to represent four broad multi-spectral wavebands according to band ranges of TM (blue – B: 450∼520 nm; green – G: 520∼600 nm; red – R: 630∼690 nm; near-infrared – NIR: 760∼900 nm). The following VIs NDVI [18], RVI [19], RB [20], VNVI, ANVI, and EVI [21] were also calculated for analysis to examine whether they can enhance spectral separability:
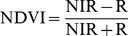
(1)

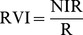
(2)

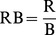
(3)

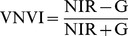
(4)

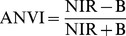
(5)

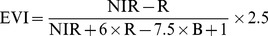
(6)


### Hyper-spectral Data Analysis

To estimate how *Spartina* is separable from *Phragmites* and *Scirpus* by using hyper-spectral remote sensing on different days, the statistical difference and separability of the hyper-spectral bands between plant species were calculated. First, the Mann-Whitney U-test was employed on the 5 nm-wide bands between every pair of vegetation types and the significance level was <0.05. The null hypothesis assumes that there is no significant difference of the same 5 nm-wide band between one type and another. If this null hypothesis was rejected, the alternative hypothesis was that they are significantly different. The significantly different bands suggested the potential to discriminate plant species, but in order to compare the discrimination ability, it’ d better to quantify how far they are in the statistical spaces (the spectral separability).

The separability of the statistically different bands was then quantified using Jeffries-Matusita (JM) distance. The JM distance between a pair of probability functions is the measure of the average distance between the density functions of two classes, indicating how successful the two classes will be discriminated [22]. The JM distance has upper and lower bounds that vary between 0 and 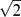
(≈1.414), with 1.414 implying classification of two classes with 100% accuracy [23]. The JM distances were calculated on each individual band without considering various band combinations.

One advantage of JM distance over other measurements of statistical separability, such as divergence, is that an upper bound of error probability for classification could be estimated [22]. According to the estimated error probability, the separability of statistically different hyper-spectral bands was classified into four categories: perfect-separability (error probability <1%), good -separability (1% ≤ error probability <7%), normal-separability (7% ≤ error probability <14%), and poor-separability (error probability ≥14%). The formula for computing the JM distance and the error probability (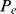) could be found in Swain and Davis [22].

### Multi-spectral Data Analysis

To estimate how *Spartina* is separable from *Phragmites* and *Scirpus* by multi-spectral remote sensing on different days, the same process as in a hyper-spectral analysis was applied to calculate the statistical difference and JM distance between each pair of plant species based on multi-spectral broad bands (B, G, R, NIR) and the commonly used VIs of NDVI, RVI, RB, VNVI, ANVI, EVI).

## Results

### The Reflectance Spectral Data Corresponding to Different Phenological Stages

**Figure 3:**
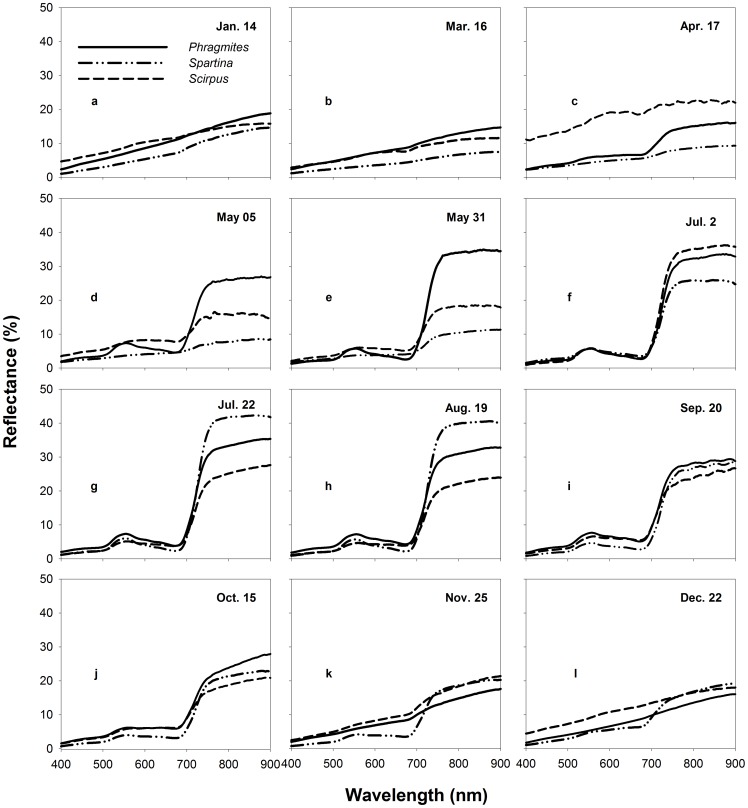
Field reflectance spectra of *Spartina*, *Phragmites*, and *Scirpus* at various phenological stages.

The mean reflectance spectral curves of *Spartina*, *Phragmites*, and *Scirpus* on different days at various phenological stages were plotted in Fig. 3. Spectral reflectance curves of the plants and the difference between them were affected by phenology. As the phenological stage changed, their spectral signatures exhibited differences in both magnitude and shape of reflectance curves. In the late-withering, dormant, and early germination stages (Fig. 3a, b, c, k, and l), the reflectance curves of all plants showed slightly rising lines with no green peak, steep red edge, or near-infrared plateaus (common characteristics of green vegetation). The low chlorophyll content at these stages alone probably can explain the lack of green peak, while the higher reflectance in red due to no chlorophyll absorption and the lower reflectance around 800 nm associated with sparse leaf/canopy structure can explain the absence of the red edge. *Phragmites* was the earliest to show typical characteristics of green vegetation (Fig. 3c). *Phragmites* began to show a steep red edge and infrared plateau from April 17 as it sprouted in early April, though no green peaks were observed. Afterwards, the significant green peak, steeper red edge, and higher near-infrared plateau began to show from May 5 when *Phragmites* grew up to about 100 cm (Fig. 3d–i). The reflectance of near-infrared began to decline and the typical characteristics of green vegetation of its reflectance curves gradually disappeared as *Phragmites* was inflorescent and withering. *Scirpus* also emerged in early April, but its rapid growth did not begin until late April; thus, its reflectance spectra showed a normal curve of green vegetation until May 5 (Fig. 3d) when it already grew to *ca.* 15 cm. In its following growth, flowering, fruit development, and even early withering stage, *Scirpus* showed higher near-infrared plateau (Fig. 3e–i). However, its reflectance in green and near-infrared wavelength regions declined quickly from August as *Scirpus* turned to mature and withering stages early. Conversely, *Spartina* generated and grew about one month later than *Phragmites* and *Scirpus*. Thus, in late spring (e.g., May 5 and 31), it has significantly lower reflectance than the *Phragmites* and *Scirpus* in green and near-infrared wavelength regions (Fig. 3d–e). *Spartina* was the latest to show a steep red edge and near-infrared plateau (Fig. 3e), but later, it shows the highest reflectance in the near-infrared region (Fig. 3g–h). On the other hand, *Spartina* withered later and slower than *Phragmites* and *Scirpus*. Thus, its canopy reflectance curves were more similar to normal curves of green vegetation in later times of a year than *Phragmites* or *Scirpus* (Fig. 3k–l).

### Hyper-spectral Statistical Difference and JM Distance Between Plants

**Figure 4:**
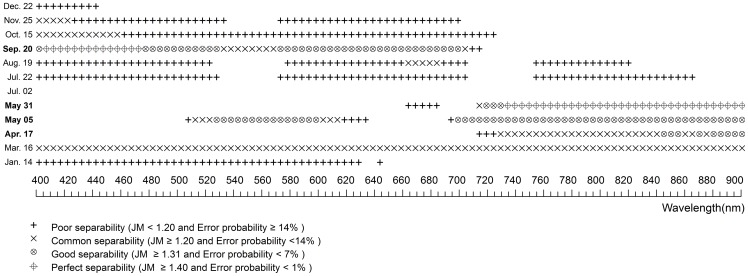
The spectral separability of hyper-spectral bands between *Phragmites* and *Spartina* at various phenological stages. The blank symbol stands for statistically similar bands, while the other symbols stand for significantly different bands with different separability determined by JM distance.

**Figure 5:**
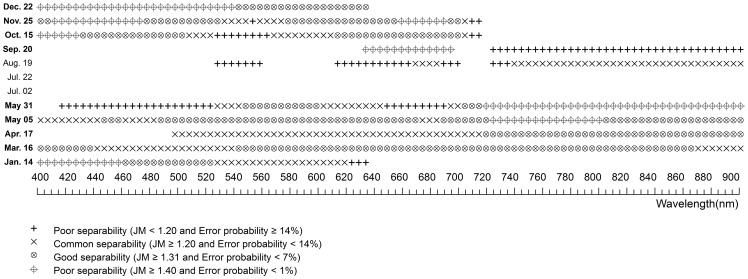
The spectral separability of hyper-spectral bands between *Scirpus* and *Spartina* at various phenological stages. The blank symbol stands for significantly similar bands, while the other symbols stand for significantly different bands with different separability determined by JM distance.

**Figure 6:**
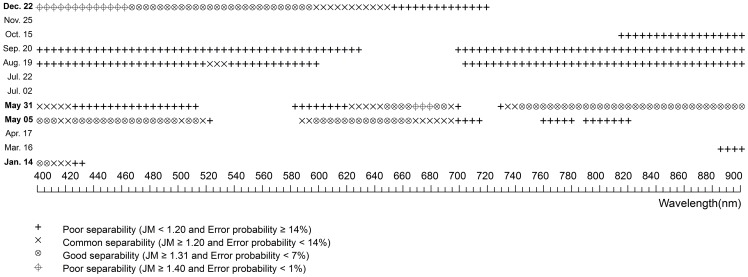
The spectral separability of hyper-spectral bands between *Scirpus* and *Phragmites* at various phenological stages. The blank symbol stands for significantly similar bands, while the other symbols stand for significantly different bands with different separability determined by JM distance.

Fig. 4, 5 and 6 shows the statistical differences and JM distances at different phenological stages on 5 nm-wide hyper-spectral bands between each pairs of species, respectively. Good- and perfect- separability between *Phragmites* and *Spartina* was achieved on September 20 and May 31. These perfect- separability bands on September 20 were between 405 and 475 nm while those on May 31 were between 735 and 900 nm. Those good- separability bands were distributed in the visible wavelength region (475–530 nm and 570–695 nm) on September 20 and in red and red edges (715–735 nm) on May 31. Good-separability bands between *Phragmites* and *Spartina* were also observed on April 17 and May 5. These bands either fell into the green wavelength region or near-infrared wavelength region. On other days, No perfect- or good- separability bands emerged between *Phragmites* and *Spartina*.

It is obviously that *Spartina* was more separable from *Scirpus* than from *Phragmites* as a whole (Comparing to Fig. 4 to 5). No statistically different bands were found on July 2, 22 and there were no good- separability bands on August 19 between *Scirpus* and *Spartina*, when they were both in stages with high chlorophyll content. Nevertheless, with the exception of July 2, July 22, and August19, there were always many statistically different bands with either perfect- separability or good- separability. The perfect-separability bands primarily distributed in blue, red, and near-infrared wavelength regions, while the good- separability bands were more widely distributed.

There were few good- or perfect- separability bands between the two native species on most days (Fig. 6). Among all sampled days, May 5 and 31 were the best for discrimination between *Scirpus* and *Phragmites*. The best bands for discrimination them were first concentrated in blue and red wavelength regions on May 5 and then shifted to red and near-infrared regions on May 31. Though December 22 showed perfect- separability and good-separability on a many bands, late December is not ideal for discrimination because the distribution of *Scirpus* was narrowed by tidal water and was unable to get the real extent of *Scirpus* according to our field observation.

### Multi-spectral Statistical Difference and JM Distance Between Plants

Tables 2, 3, and 4 show the statistical differences and JM distances on multi-spectral bands (B, G, R, and NIR) and the commonly used VIs at the multi-phenological stages between *Spartina* and *Phragmites*, *Spartina* and *Scirpus*, and *Phragmites* and *Scirpus,* respectively. Excluding VIs, spectral difference and separability were limited on broad multi-spectral bands. *Spartina* and *Phragmites* had perfect-separability on May 31 (NIR) and good separability on May 5 (G, R, and NIR) and September 20 (B, G, and R) (Table 2). The broadband spectral separability between *Spartina* and *Scirpus* was much better than that between *Spartina* and *Phragmites*. Perfect- or good-separability bands were found on all surveyed days except July 2, July 22, and August 19 when *Spartina* and *Scirpus* were both near their growth summit (Table 3). The three tables also show that the VIs could enhance the spectral separability between the plants at many stages. The four broad bands between *Spartina* and *Phragmites* on November 25 and December 22 had either poor- separability or were not statistically different, but the VIs like NDVI, RVI, VNVI, and ANVI were ranked with good- or perfect- separability (Table 2). The VIs significantly enhanced the spectral separability between *Spartina* and *Scirpus* on January 14, April 17, and July 22 (Table 3). The VIs also made it possible to discriminate *Phragmites* and *Scirpus* in early winter and mid spring, as perfect-separability was obtained on the VIs, like NDVI and ANVI, on January 14 and April 17 (Table 4). On the whole, NDVI, VNVI, ANVI, and RVI more frequently performed enhanced separability than their combined original broad bands.

**Table 2:** The multi-spectral separability between *Phragmites* and *Spartina* at various phenological stages.

Date	*Spartina vs. Phragmites*									
	B	G	R	NIR	NDVI	RVI	RB	VNVI	ANVI	EVI
Jan. 14	–	–	–	#	+	+	++	+	++	#
Mar. 16	+	+	+	+	–	–	#	#	#	–
Apr. 17	#	#	#	–	–	–	#	–	–	++
**May 5**	–	++	#	++	++	++	–	++	++	++
**May 31**	#	–	–	+++	+++	+++	+++	+++	+++	+++
Jul. 2	#	#	#	+	–	–	#	#	+	–
Jul. 22	–	#	–	–	–	+	–	+	+	–
Aug. 19	–	#	#	–	–	–	#	++	++	–
**Sep. 20**	++	++	++	#	–	–	#	++	+	#
Oct. 15	–	–	–	#	–	–	#	–	–	#
Nov. 25	–	–	–	#	+++	+++	–	+++	+++	+
Dec. 22	–	#	–	#	++	++	–	++	++	++

–: poor separability (error probability≥14%);

+: normal separability (14% ≥error probability ≥7%);

++: good separability (7% ≥error probability ≥1%);

+++: perfect separability (error probability ≤1%);

#: no significant difference.

Dates with good or perfect separability bands were highlighted in bold.

**Table 3:** The multi-spectral separability between *Spartina* and *Scirpus* at various phenological stages.

Date	*Spartina vs. Scirpus*									
	B	G	R	NIR	NDVI	RVI	RB	VNVI	ANVI	EVI
**Jan. 14**	++	+	–	#	+++	+++	+++	+++	+++	+
**Mar. 16**	+	++	++	++	–	–	#	–	–	#
**Apr. 17**	–	–	–	–	+++	+	+	+++	++	–
**May 5**	++	++	++	++	#	#	#	#	#	++
**May 31**	–	++	–	+++	++	++	#	–	+	+++
Jul. 2	#	#	#	#	#	#	#	#	#	#
Jul. 22	#	#	+	+	+	++	+++	–	–	+
Aug. 19	#	#	#	+	–	–	#	+	–	–
Sep. 20	++	++	+++	#	++	++	++	++	+	–
**Oct. 15**	++	+	++	#	++	++	–	++	++	–
**Nov. 25**	++	+	+++	#	+++	+++	–	+++	+++	++
**Dec. 22**	+++	++	++	#	+++	+++	+++	+++	+++	+++

–: poor separability (error probability≥14%);

+: normal separability (14% ≥error probability ≥7%);

++: good separability (7% ≥error probability ≥1%);

+++: perfect separability (error probability ≤1%);

#: no significant difference.

Dates with good or perfect separability bands were highlighted in bold.

**Table 4:** The multi-spectral separability between *Phragmites* and *Scirpus* at various phenological stages.

Date	*Phragmites vs. Scirpus*									
	B	G	R	NIR	NDVI	RVI	RB	VNVI	ANVI	EVI
Jan. 14	–	#	#	#	+++	+++	+	+++	+++	–
Mar. 16	#	#	#	–	–	–	–	–	–	–
Apr. 17	–	–	–	–	+++	+	++	+++	++	++
**May 5**	++	#	++	–	++	+	–	++	++	+
**May 31**	–	#	++	++	+++	+++	+++	+++	+++	+++
Jul. 2	#	#	#	#	#	#	++	#	#	#
Jul. 22	–	–	#	–	#	#	–	#	#	–
Aug. 19	–	–	#	–	#	#	#	#	#	–
Sep. 20	–	#	#	#	#	#	–	#	#	#
Oct. 15	#	#	#	–	–	–	–	–	#	–
Nov. 25	#	#	#	#	#	#	#	#	#	–
**Dec. 22**	++	++	–	#	+++	+++	++	+++	+++	#

–: poor separability (error probability≥14%);

+: normal separability (14% ≥error probability ≥7%);

++: good separability (7% ≥error probability ≥1%);

+++: perfect separability (error probability ≤1%);

#: no significant difference.

Dates with good or perfect separability bands were highlighted in bold.

### The Best Dates and Bands to Discriminate Spartina

Tables 5 and 6 summarized the comprehensive information to determine the best periods for discriminating *Spartina* from *Phragmites* and *Scirpus*. Among all our sampled days, May 5, 31 and September 20 (which represent two periods, *e.g.* later spring and early autumn) are clearly the best to discriminate *Spartina*, as they have good- or perfect- separability between *Spartina* and either of the other two natives on either hyper- or multi-spectral bands. However, hyper-spectral bands clearly show priority to detect more minor differences by providing more narrow bands that could discriminate each plant from the others at more periods, and the commonly used VIs based on multi-spectral broad bands could enhance their ability for plant discrimination, in terms of expanding time periods and increasing separability on original bands (Tables 5 and 6).

**Table 5:** Hyper-spectral bands (wavelengths in nm) with good or perfect separability (error probability ≤7%) between dominant plants at different phenological stages.

Date	A	B	C1	C2
Jan. 14				400–530
Mar. 16				400–455 530–870
Apr. 17		785–815 825–900		710–785 815–820
**May 5**		520–605 695–900		435–520 405–675 685–695
**May 31**	740–900	715–740		540–605 700–715
Jul. 2		875–885		705–875 885–900
Jul. 22				470–685
Aug. 19				755–790
**Sep. 20**		405–520 565–700	520–530	700–705
Oct. 15				400–510 605–700
Nov. 25				400–530 575–655 655–700
Dec. 22				400–695

A: the bands have good or perfect separability between each pair among *Spartina*, *Phragmites*, and *Scirpus*; B: the bands have good or perfect separability between *Spartina* and *Phragmites* and between *Spartina* and *Scirpus*; C1: the bands have good or perfect separability only between *Spartina* and *Phragmites*, C2: the bands have good or perfect separability only between *Spartina* and *Scirpus*. The best dates for *Spartina* discrimination are highlighted in bold.

**Table 6:** Multi-spectral bands and VIs with good or perfect separability (error probability ≤7%) between dominant plants at different phenological stages.

Date	A	B	C1		C2
Jan. 14		RB ANVI		**B** NDVI RVI VNVI	
Mar. 16				**G R NIR**	
Apr. 17			EVI	NDVI VNVI ANVI	
**May 5**		**G NIR** EVI	NDVI RVI VNVI ANVI	**B R**	
**May 31**	**NIR** NDVI, RVI EVI		RB VNVI ANVI	**G**	
Jul. 2					
Jul. 22				RVI RB	
Aug. 19			VNVI ANVI		
**Sep. 20**		**B G R** VNVI		NDVI RVI RB	
Oct. 15				**B R** NDVI RVI VNVI ANVI	
Nov. 25		NDVI RVI VNVI ANVI		**B R** EVI	
Dec. 22		NDVI RVI VNVI ANVI EVI		**B G R** RB	

A: the bands and VIs have good or perfect separability between each pair among *Spartina*, *Phragmites*, and *Scirpus*; B: the bands and VIs have good or perfect separability both between *Spartina* and *Phragmites* and between *Spartina* and *Scirpus*; C1: the bands and VIs have good or perfect separability only between *Spartina* and *Phragmites*, C2: the bands and VIs have good or perfect separability only between *Spartina* and *Scirpus*. The best dates for *Spartina* discrimination are highlighted in bold.

## Discussion

### Spectral Characteristics and Separability Affected by their Phenological Stages

The spectral characteristics of each species were not unique between phenological stages. They were varying significantly according to their phenological stages. The phenology of plants generally determines the spectral characteristics of vegetation by determining the canopy structure, growth stage, water content, and chlorophyll content of vegetation [24]. Spectral differences near the green waveband peak (around 550 nm), red edge (around 720 nm), and in the near-infrared plateau are considered important for discriminating vegetation types at growing stages [4]. The differences near red edge and in the near-infrared plateau are usually characterized by differences in canopy structure and the differences near the green waveband peak are usually characterized by differences in chlorophyll content [11]. In this case, the canopy structure between *Spartina* and *Phragmites* was more similar than that between *Spartina* and *Scirpus* in most phonological stages (see Figure S1), explaining why *Spartina* is more spectrally separable from *Phragmites* than *Scirpus*. *Spartina* generates later than *Phragmites* and *Scirpus*, causing it to have notably less green vegetative amounts and low plant height in late spring, thus making late spring the best period to monitor *Spartina* by increasing the spectral separability at wavelengths near the green waveband peak, red edge, and in the near-infrared plateau (see Tables 5 and 6). In summer, the three plants reached their peak heights with high chlorophyll content and similar canopy structures (see Figure S1). Thus, they were difficult to separate with any hyper−/multi-spectral bands. A previous study, in a single summer, demonstrated that the vegetation amount in terms of the height and cover was important in determining the levels of reﬂectance, particularly at the near-infrared band of saltmarsh communities [25]. In this study, *Spartina* showed average high levels of near-infrared reflectance in summer (Fig. 3g and 3 h), as it had denser covers than *Phragmites* and *Scirpus*, as well as clearly higher canopy altitudes than *Scirpus* (see Figure S1), but this average high near-infrared reflectance was not statistically significant. Early autumn, i.e. the flowering stage of *Spartina*, is another good period to discriminate *Spartina*. The bloom of *Spartina* not only changed its pigment content and proportion but also changed its canopy structure suddenly, while *Phragmites* was at a vegetative growth or inflorescence stage and *Scirpus* was at a withering stage (see Figure S1), making perfect separability between *Spartina* and the other two on bands located at the visible wavelength range. Another phenological characteristic of *Spartina* is that it senesces and withers more slowly than *Phragmites* and *Scirpus*; thus, in early winter, *Spartina* can preserve more green vegetative parts (Figure S1). This phenomenon can result in statistical spectral differences in the visible wavelength region between *Spartina* and the other plants (Fig. 4 and 5). Though these differences were small between *Spartina* and *Phragmites* probably because of their similar canopy structure and tended to be marked out in broad multi-spectral bands, they can be enhanced by specific VIs to have enough separability to distinguish them (Table 2).

### Look into the Timing of Saltmarsh Vegetation Classification

**Figure 7:**
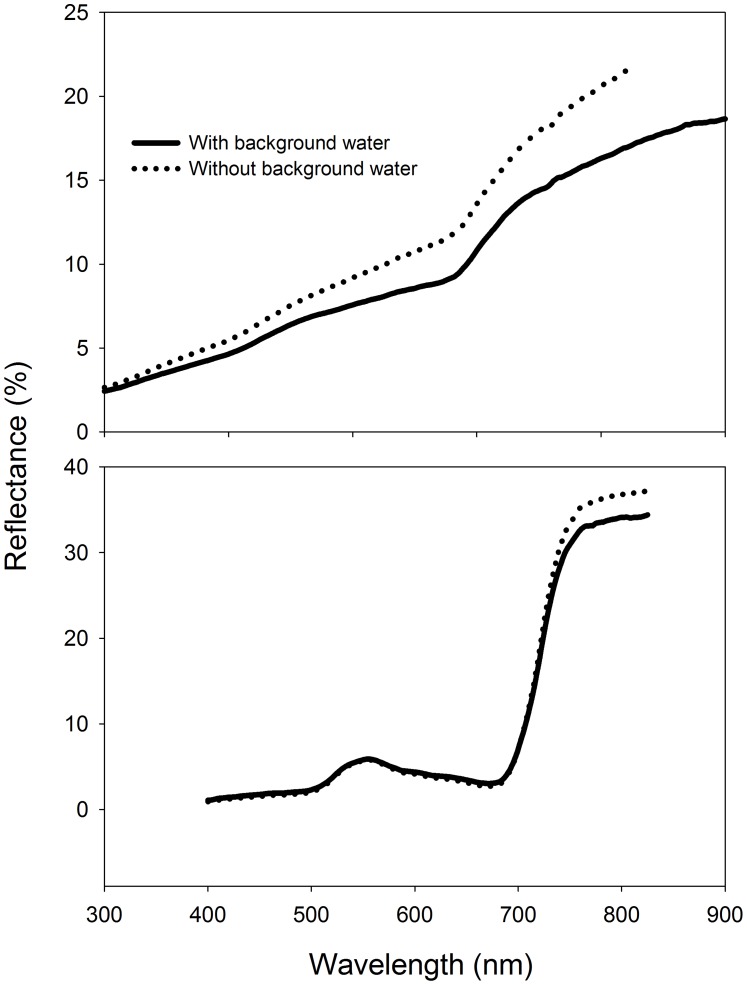
Field reflectance spectra of *Scirpus* without/with background water (*ca.* 5cm in depth). The left frame is sampled on October 15 and the right one on July 2.

Limited by the weather, tides, and expensive cost of filed work at this saltmarsh, we were not be able to collect spectra daily, weekly, or even bi-weekly. However, with samples collected on those 12 single days (table 1), we can still infer the best periods for classification based on the fact that these vegetation change phonological stage gradually. This study clearly demonstrates that May 5, May 31, and September 20 achieved the best separability to classify or mapping distribution of *Spartina* in the Yangtze River Estuary. The corresponding phonological stages are the germination, early vegetative growth, and flowering stages of *Spartina*, which are about in late spring and early autumn of a year (Table 7). The withering stage of *Spartina* (late autumn and early winter of a year, represented by samples collected on November 25 and December 22) may also be a good period for mapping its spatial extent; however, it may need to take advantage of proper VIs. Yet, this late-autumn or early winter period should never be good timing for *Scirpus* monitoring because aboveground biomass has been washed away by tidal water and its spectra mixes with background water and mud (Figure S1). In an ecology view, *Scirpus* is as important as *Spartina* to be monitored because it is more preferred by waterfowls but has been forced to populate a narrow zone close to the sea due to competitive disadvantage [3]. As a result, it is even more difficult to map *Scirpus* by remote sensing as the tidal waters regularly influence its relatively short and sparse canopy. We collected *Scirpus* spectra samples in some depression plots where water remains after the tide and found that its spectral characteristic was much different than that without a water background at its early growing and withering stages, but not its peak growing stage (Fig. 7). Moreover, our analysis has suggested that the peak growing stage is the worst timing for vegetation discrimination. Thus, it is very important to consider timings with minimum tidal water influence for *Scirpus* monitoring. The information provided here would be helpful to collect, process, and classify images by guiding investigators toward suitable periods for dynamically monitoring *Spartina* as well as the other two saltmarsh species. Some of our previous studies that validated the proposed periods are qualified for *Spartina* monitoring in the Yangtze River Estuary [26], [27]. However, as the spectra were collected from roughly 0.5 m square canopy areas, it would be difficult to directly extrapolate these reflectance signatures to real canopy cover reflectance captured by a remote sensor.

**Table 7:** Compiled information for *Spartina* discrimination.

Best dates among all sampled dates	Best separable hyper-spectral bands	Best separable multi-spectral bands	The phenology representedby the dates	The approximatedseason
May 5	520–605 nm and 695–900 nm	G NIR	Germination	Late spring
May 31	715–900 nm	NIR	early vegetative growth	Late spring
Sep. 20	405–520 nm and 565–700 nm	B G R	flowering	Early autumn

This tables summarized the best timings and the corresponding spectral bands for *Spartina* discrimination based on our results listed in tables 2–6, and figures 4–6.

While phenology is critical in determining vegetation spectral characteristics, the timings of phenological stages are adjusted to climate, like temperature and precipitation patterns. Therefore, optimum periods for saltmarsh vegetation discrimination might shift between years and the right way to determine the periods is to investigate the suggested emergence of phonological divergence between plants but to not adhere to any predetermined periods. A strategy that integrates multi-phenological images may produce a better result. However, improper usage of multi-date images might also waste money, produce redundant information, and increase image processing effort. Still, based on the abundant information provided, users can have their choice based on effectiveness and cost.

### Conclusion

The field spectra of saltmarsh vegetation including *Spartina*, *Phragmites*, and *Scirpus* were collected and analyzed at multi-phenological stages in the largest estuarial wetland of the Yangtze River Estuary to find the best periods for mapping *Spartina*. The three plants showed changing spectral characteristics affected by their phenological stages. The results suggest that the germination and early growth stages of *Spartina* (late spring) as well as its flowering stage (early-autumn) may be the best periods to discriminate *Spartina* from *Phragmites* and *Scirpus* (Table 7). The corresponding best-separable hyper−/multi-spectral bands were also determined for each period (Table 7). In late-spring, the most separable hyper-spectral bands are located near the green waveband peak, red edge, and in the near-infrared plateau and the multi-spectral bands are NIR and G. In early-autumn, the most separable hyper-spectral bands are located at the whole visible region and the multi-spectral bands are B, G, and R. By utilizing VIs like NDVI, ANVI, VNVI, and RVI, which take advantage of the reflectance contrast between NIR and other bands, it is also possible to discriminate *Spartina* at its withering stage (late autumn and early winter). Proper usage of specific VIs might also enhance the spectral separability between those saltmarsh species in other periods.

## Supporting Information

Figure S1 **Photographs of the saltmarsh species at different times in Dongtan.** The phenology of them can be observed. Those on the left are *Phragmites*, the middle are *Spartina*, and the right are *Scirpus*.
(DOCX)

## References

[1] Zhao B, Li B, Zhong Y, Nakagoshi N, Chen JK (2005) Estimation of ecological service values of wetlands in Shanghai, China. Chin Geogr Sci 15: 151–156.

[2] Chen ZY, Li B, Zhong Y, Chen JK (2004) Local competitive effects of introduced Spartina alterniflora on Scirpus mariqueter at Dongtan of Chongming Island, the Yangtze River estuary and their potential ecological consequences. Hydrobiologia 528: 99–106.

[3] Li B, Liao CH, Zhang XD, Chen HL, Wang Q, et al (2009) Spartina alterniflora invasions in the Yangtze River estuary, China: An overview of current status and ecosystem effects. Ecol Eng 35: 511–520.

[4] Adam E, Mutanga O, Rugege D (2010) Multispectral and hyperspectral remote sensing for identification and mapping of wetland vegetation: a review. Wetl Ecol Manag 18: 281–296.

[5] Rosso PH, Ustin SL, Hastings A (2005) Mapping marshland vegetation of San Francisco Bay, California, using hyperspectral data. Int J Remote Sens 26: 5169–5191.

[6] Artigas FJ, Yang JS (2005) Hyperspectral remote sensing of marsh species and plant vigour gradient in the New Jersey Meadowlands. Int J Remote Sens 26: 5209–5220.

[7] Thomson AG, Huiskes A, Cox R, Wadsworth RA, Boorman LA (2004) Short-term vegetation succession and erosion identified by airborne remote sensing of Westerschelde salt marshes, The Netherlands. Int J Remote Sens 25: 4151–4176.

[8] Hochberg EJ, Andrefouet S, Tyler MR (2003) Sea surface correction of high spatial resolution Ikonos images to improve bottom mapping in near-shore environments. IEEE T Geosci Remote 41: 1724–1729.

[9] Daughtry CST, Walthall CL (1998) Spectral discrimination of Cannabis sativa L. leaves and canopies. Remote Sens Environ 64: 192–201.

[10] Cochrane MA (2000) Using vegetation reflectance variability for species level classification of hyperspectral data. Int J Remote Sens 21: 2075–2087.

[11] Schmidt KS, Skidmore AK (2003) Spectral discrimination of vegetation types in a coastal wetland. Remote Sens Environ 85: 92–108.

[12] Pena-Barragan JM, Lopez-Granados F, Jurado-Expoosito M, Garcia-Torres L (2006) Spectral discrimination of Ridolfia segetum and sunflower as affected by phenological stage. Weed Res 46: 10–21.

[13] Koger CH, Shaw DR, Watson CE, Reddy KN (2003) Detecting late-season weed infestations in soybean (Glycine max). Weed Technol 17: 696–704.

[14] Hansen PM, Schjoerring JK (2003) Reflectance measurement of canopy biomass and nitrogen status in wheat crops using normalized difference vegetation indices and partial least squares regression. Remote Sens Environ 86: 542–553.

[15] Best EPH, Zippin M, Dassen JHA (1981) Growth and production ofPhragmites australis in Lake Vechten (the Netherlands). Aquat Ecol 15: 165–173.

[16] Ma ZJ, Li B, Zhao B, Jing K, Tang SM, et al (2004) Are artificial wetlands good alternatives to natural wetlands for waterbirds? A case study on Chongming Island, China. Biodivers Conserv 13: 333–350.

[17] Yan Y-E, Guo H-Q, Gao Y, Zhao B, Chen J-Q, et al (2010) Variations of net ecosystem CO2 exchange in a tidal inundated wetland: Coupling MODIS and tower-based fluxes. J Geophys Res-Atmos 115: D15102.

[18] Rouse JW, Haas RH, Schell JA, Deering DW (1973) Monitoring vegetation systems in the Great Plains with ERTS. Third ERTS Symposium. Washington, D.C: NASA. 309–317.

[19] Jordan CF (1969) Derivation of leaf-area index from quality of light on forest floor. Ecology 50: 663–666.

[20] Everitt JH, Villarreal R (1987) Dectecting huisache (Acacia farnesiana) and Mexican palo-verde (Parkinsonia aculeata) by aerial photography. Weed Sci 35: 427–432.

[21] Huete A, Didan K, Miura T, Rodriguez EP, Gao X, et al (2002) Overview of the radiometric and biophysical performance of the MODIS vegetation indices. Remote Sens Environ 83: 195–213.

[22] Swain PH, Davis SM (1978) Remote Sensing: the Quantitative Approach. New York: McGraw-Hill. 396 p.

[23] Richards JA (1993) Remote Sensing Digital Image Analysis: an Introduction. Berlin; New York: Springer-Verlag. 340 p.

[24] Pu RL, Gong P (2000) Hyperspectral Remote Sensing and Its Applications. Beijing: Higher Education Press. 254 p.

[25] Gao ZG, Zhang LQ (2006) Identification of the spectral characteristics of natural saltmarsh vegetation using indirect ordination: a case study from Chongming Island, Shanghai, China. Chin J Plant Ecol 30: 252–260.

[26] He M-M, Zhao B, Ouyang Z-T, Yan Y-E, Li B (2010) Linear spectral mixture analysis of Landsat TM data for monitoring invasive estuarine vegetation. Int J Remote Sens 31: 4319–4333.

[27] Ouyang Z-T, Zhang M-Q, Xie X, Shen Q, Guo H-Q, et al (2011) A comparison of pixel-based and object-oriented approaches to VHR imagery for mapping saltmarsh plants. Ecol Inform 6: 136–146.

