# Supplementary material for: Spectral Discrimination of the Invasive Plant Spartina alterniflora at Multiple Phenological Stages in a Saltmarsh Wetland
Source: PLoS One. 2013 Jun 27;8(6):e67315. doi: 10.1371/journal.pone.0067315 (PMC3695028; doi:10.1371/journal.pone.0067315)

**Figure S1: Photographs of the saltmarsh species at different times in Dongtan.** The phenology of them can be observed. Those on the left are *Phragmites*, the middle are *Spartina*, and the right are *Scirpus*. The individuals in this figure have given written informed consent, as outlined in the PLOS concern form, to publication of their photograph.


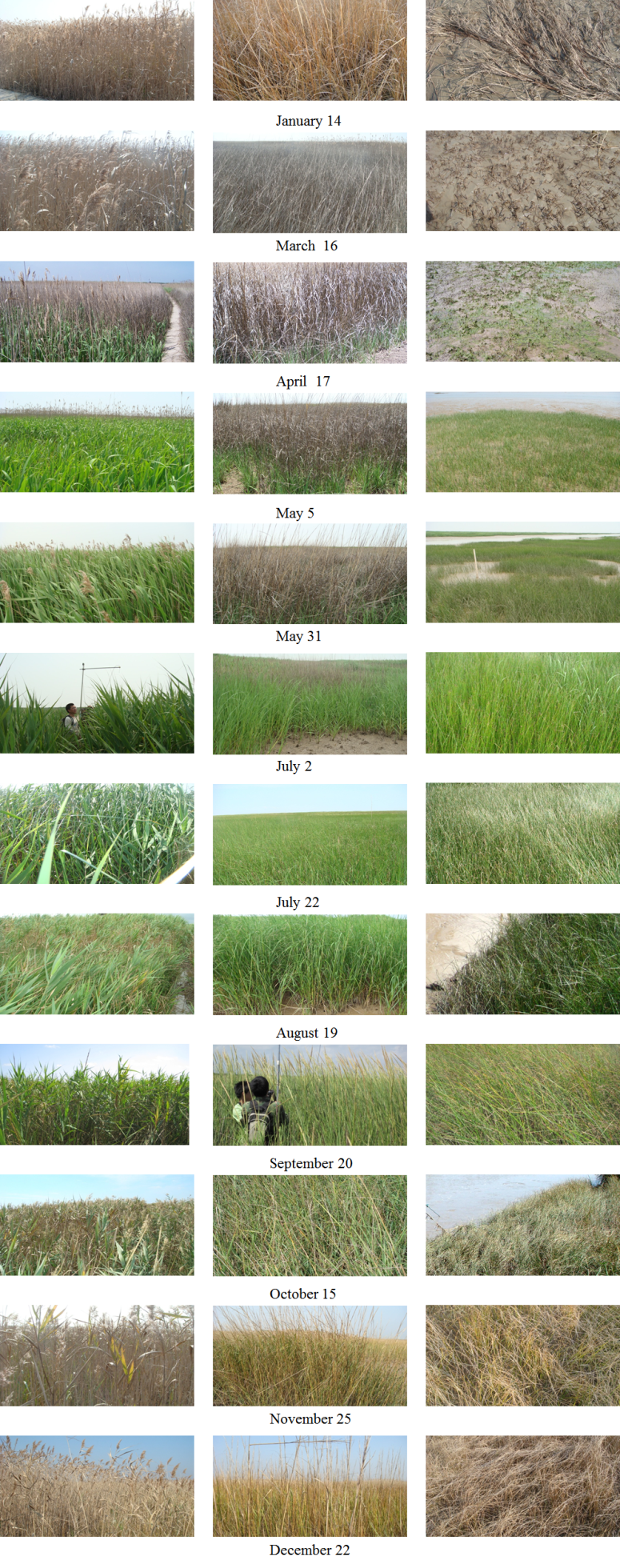

Supplement: Figure S1 — Photographs of the saltmarsh species at different times in Dongtan. The phenology of them can be observed. Those on the left are Phragmites, the middle are Spartina, and the right are Scirpus. (DOCX) [file pone.0067315.s001.docx]
